# Treatment Status of Patients with B-Thalassemia Major in Northern Iran: Thalassemia Registry System

**Published:** 2019-07

**Authors:** Mehrnoush KOSARYAN, Hossein KARAMI, Hadi DARVISHI-KHEZRI, Rosetta AKBARZADEH, Aily ALIASGHARIAN, Khadijeh BROMAND

**Affiliations:** 1. Department of Pediatric, Thalassemia Research Center, Hemoglobinopathy Institute, Mazandaran University of Medical Sciences, Sari, Iran; 2. Student Research Committee, Thalassemia Research Center, Hemoglobinopathy Institute, Mazandaran University of Medical Sciences, Sari, Iran; 3. Thalassemia Research Center, Hemoglobinopathy Institute, Mazandaran University of Medical Sciences, Sari, Iran

**Keywords:** Beta thalassemia major, Intermedia thalassemia, Deferoxamine, Deferiprone, Deferasirox

## Abstract

**Background:**

Electronic registry system of beta-thalassemia patients was run by Thalassemia Research Center (TRC) in 2017. The aim of the current study was presentation of therapeutic status in these patients at Mazandaran Province, Iran.

**Methods:**

Therapeutic status variables including: Name of cities and hospitals, age and sex of patients, dependent and non-transfusion-dependent, starting age of the blood transfusion and iron-chelating agents, blood group and Rh, washed blood transfusion, abnormal antibody, transfusion reactions, mean hemoglobin during the last 3 months, type of iron chelators, iron chelators dosage, serum ferritin, and the use of hydroxyurea.

**Results:**

Overall, 1831 patients were registered [891 male (48.7%)]. Mean age of patients was 30±9.7 yr. Average of hemoglobin levels for female and male were 9.1±5.1 and 9.4±6.3 gr/dl, respectively. Seventy-six percent of transfusion-dependent patients (1385) have received iso-group PRBC (packed red blood cells), after crossmatch. The most common blood group among patient was type O-positive (35.7%). Monotherapy with desferrioxamine was most type of used iron-chelating agent in these patients (47.2%). Mean of ferritin was 3300±7800 (ng/ml). Twenty-eight percent of patients (484) have received hydroxyurea; proportion of male and female was approximately equal. T2 weighted magnetic resonance imaging (MRIT2*) was measured in 62.2% of patients. Moderate and severe hepatosiderosis was 10.1% and 2.9%, respectively. Patients with moderate and severe cardiac siderosis were 11% and 5%, respectively.

**Conclusion:**

Registry findings are valuable for treatment management and ensuring patients medications. It will also provide accessibility to various levels of patients’ information for health care managers and experts to help them make appropriate decisions.

## Introduction

The main problem for patients with thalassemia major is a severe and progressive anemia that usually begins a few months after birth and may cause the death of the struggling individual (1). Stem cell transplantation is a definitive treatment, which is not feasible for all patients, and most patients are given repeated blood transfusion. The goal of the treatment is to provide sufficient hemoglobin levels to sustain life and prevent bone deformities and also to provide natural growth for the individual (1). In addition to blood transfusion, other therapeutic measures include iron-chelating agents, splenomegaly treatment, treatment of impaired growth, and treatment of cardiac and endocrine problems (1, 2).

The need for transfusion depends on the severity of anemia in patients. Some patients do not require frequent transfusions to survive; their hemoglobin level without transfusion is usually up to 8 g/dl or even higher. This group was previously called intermedia thalassemia but is currently considered as non-transfusion dependent thalassemia major (1, 2). The treatment for these patients has its own complexity, because of several complications such as growth disorder and facial deformation, enlargement of the spleen, extramedullary hematopoiesis, gallstone, osteoporosis, increased pulmonary artery pressure and events caused by hypercoagulable state in patients. Therefore, in both groups of patients, the treatment is complex and requires the cooperation of a large number of medical and dental specialists (3–7).

Thalassemia wards of northern Iran (Mazandaran Province) have been formed gradually in the big cities and then in small towns since the early 1981s and attempts were made to store medical records for patients in the nearest public hospital. Recording, monitoring and evaluating registry system are one of the remarkable developments in recent years, had a significant impact on the performance of management systems. Establishing a data management and thalassemia treatment system is one of the requirements of the countries with high prevalence of thalassemia and will be used to follow up and monitor the status of patients by clinicians.

The electronic registry system of thalassemia major patients (thalassemia registry) in northern Iran began in spring of 2016. Considering the importance of the patient’s registry system and the knowledge of physicians, medical staff and authorities in this regard, this study aimed to report the treatment status of thalassemic patients at the end of 2016.

## Methods

Approval of the Vice Chancellor of Research and Technology of Mazandaran University of Medical Sciences was obtained for the study.

The web-based application of THRegistry (Mazandaran Thalassemia Registry) was designed based on the Net Framework platform in VB.Net and the SQL Server2014 database at thr.mazums.ac.ir. This application works on 3 levels of patients, registration users and reporting users. This system with 100 variables shows the demographic, clinical, laboratory and therapeutic status of patients under the coverage of Mazandaran University of Medical Sciences (MUMS).

Patients’ treatment status includes: name of the city and hospital, age and gender of patients, blood transfusion dependency, age of transfusion, blood group and Rh, use of washed RBC, abnormal antibody, blood transfusion reactions, mean hemoglobin in the last 3 months, age of start of iron chelators, dose of iron chelators, serum ferritin, splenectomy history, use of various drugs (folic acid, aspirin, penicillin, calcium supplement and vitamin D and etc.) as well as type of health insurance. Moreover, the use of hydroxyurea as the only fetal hemoglobin (HbF) inducer drug was documented and reported in these patients. Different types of Iranian or foreign iron chelators were recorded in the same name as the generic name. The iron deposition status was measured in a graded form (normal-mild-moderate-severe) in the liver and heart using MRIT2* method (T2 weighted magnetic resonance imaging) at a center and using a device (8). The system’s ability was used to calculate mean and standard deviation, frequency and percent frequency, and plot the graph for data representation.

## Results

**Fig. 1:**
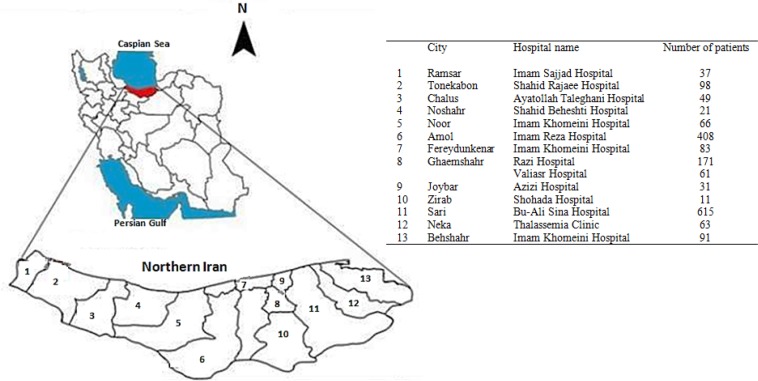
Distribution of hospitals with thalassemia wards affiliated to Mazandaran University of Medical Sciences (MUMS) and number of registered patients, 2016

In 13 counties of 22 counties in Mazandaran Province, there are clinics and thalassemia departments under the supervision of Mazandaran University of Medical Sciences (MUMS) (two centers in Ghaemshahr City). The thalassemia department of the Amirkola hospital is under the supervision of the Babol University of Medical Sciences (BUMS) and the patients are not registered in this system. Each ward is located in a general hospital category (Fig. 1).

**Fig. 2:**
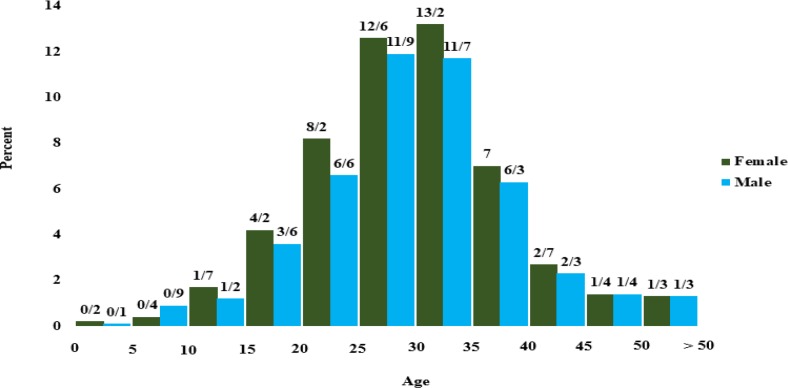
Distribution of 1385 transfusion-dependent patients by age and gender, Mazandaran Province, 2016

**Fig. 3:**
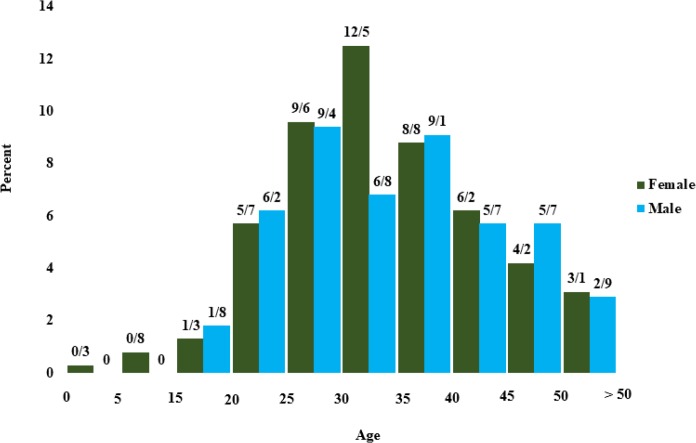
Distribution of 446 transfusion-dependent patients according to age and gender, Mazandaran Province, 2016

There are 1–3 physicians in each center. Overall, 24 physicians, including a pediatric hematologist and oncologist, an endocrinologist, 17 pediatricians, 3 internal specialists and 2 general practitioners are working in these wards. Up to the time of reporting, 1831 patients, including 943 (51.5%) women and 888 (48.5%) men were enrolled with a mean age of 30±9.7 yr (equal in both genders). All of them have health insurance coverage. Totally, 1385 patients (76%) were dependent on blood transfusion, and distribution of age and gender were shown in Fig. 2. Overall, 446 (24%) are not blood-dependent, and their age distribution according to gender is shown in Fig. 3.

**Fig. 4:**
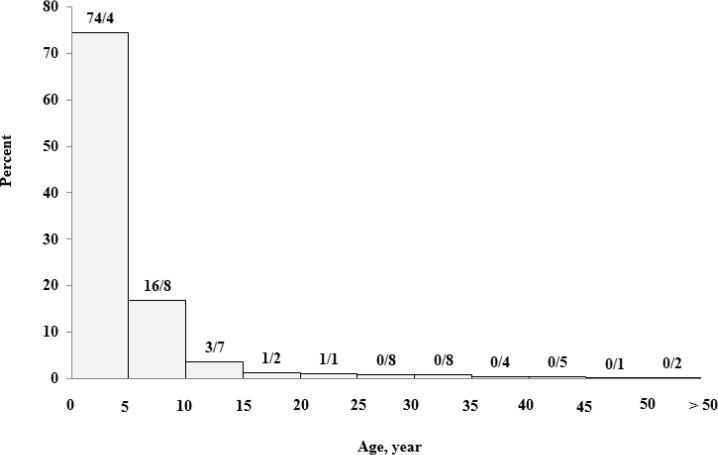
Distribution of 1385 patients with β-thalassemia major in terms of transfusion age, Mazandaran Province, 2016

In all age groups, there are many similarities between men and women, in terms of number except for the age group of 30-35 yr in non-transfusion-dependent patients, in which are women population reached almost half that of men (Fig. 3). The transfusion age was under the age of 5 in 1355 (74%) patients (Fig. 4).

**Fig. 5:**
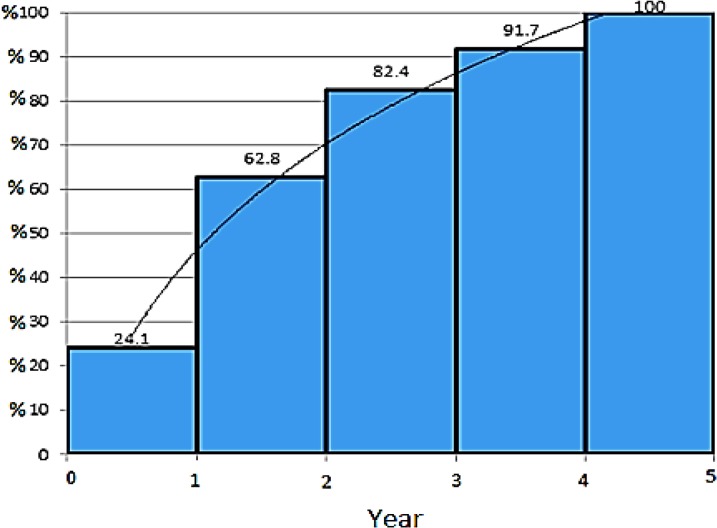
Cumulative frequency of age of transfusion in 1385 patients with β-thalassemia major – Mazandaran Province-2016

The cumulative frequency for the age of first blood transfusion in these individuals suggests that more than half of these people began to receive blood before the age of 2 (Fig. 5).

**Fig. 6:**
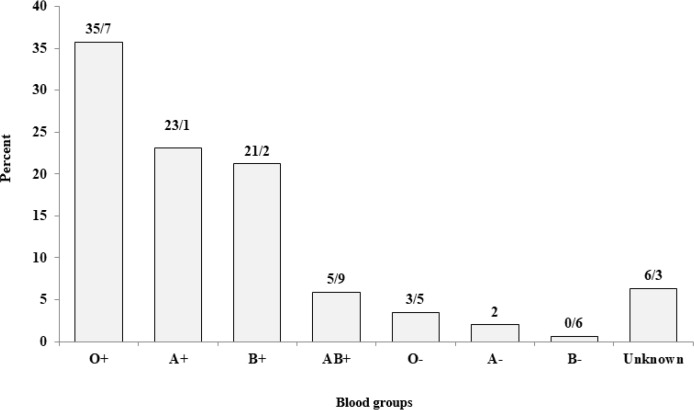
Distribution of 1831 β-thalassemia patients (major and intermedia) according to blood groups and Rh, Mazandaran Province, 2016

**Fig. 7:**
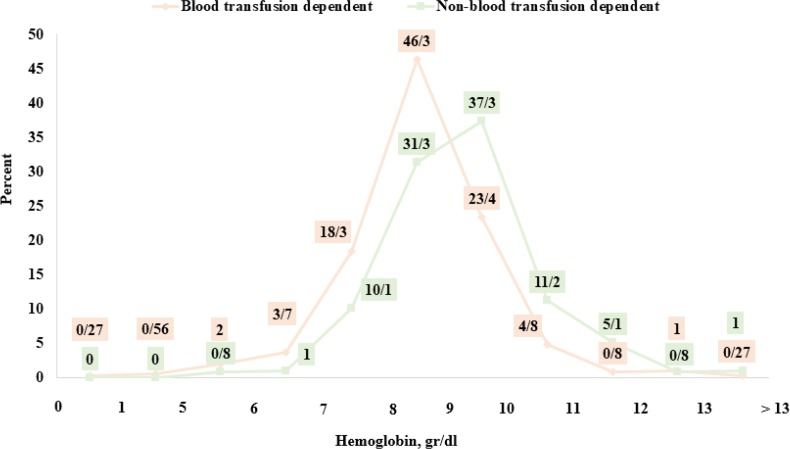
Distribution of 1831 β-thalassemia patients (major and intermedia) in terms of hemoglobin amounts and transfusion dependency, Mazandaran Province, 2016

The type of blood received in all patients is the isolated isotropic RBC after the cross matching, passed through a filter of leukocyte in either the pre-storage filtering before transfusion (leukodepleted or leukoreduced). The most frequent blood group was O^+^ group with the least frequency of 35.7% (Fig. 6). Hemoglobin levels are shown in Fig. 7 in terms of transfusion dependency.

**Fig. 8:**
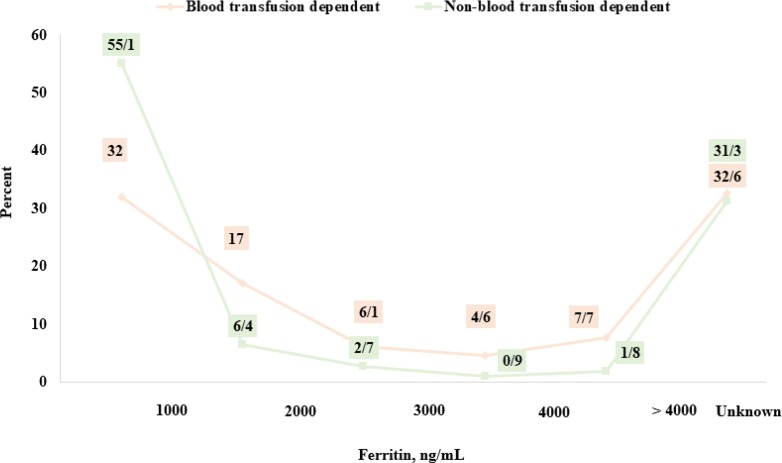
Distribution of 1831 patients with beta-thalassemia (dependent and non-dependent transfusion) in terms of serum ferritin and based on transfusion dependency, Mazandaran Province, 2016

**Fig. 9:**
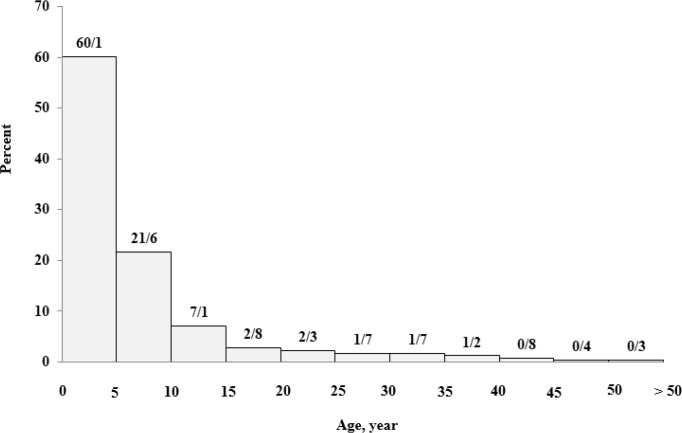
Distribution of 1831 patients with beta-thalassemia (dependent and non-dependent blood transfusion) based on the age of start of iron chelators, Mazandaran Province, 2016

In the whole Mazandaran Province, early reactions to blood transfusions washed RBC and abnormal antibodies were recorded in 10%, 5%, less than 2% of patients, respectively. The history of splenectomy was recorded in 842 patients (48.8%). Overall, 608 patients (35.2%) did not undergo the splenectomy process, and the medical records were unclear in this regard in 275 cases (16%). The mean serum ferritin concentration was 3300±7800ng/ml, and its distribution, based on transfusion dependency, is shown in Fig. 8. The mean age of the onset of iron chelators is 7.4 ± 8.9 yr and its distribution is shown in Fig. 9.

Overall, 1478 patients are taking iron chelators. The most frequently used iron chelators are one type of deferoxamine used in 1004 people (deferoxamine: 552 people, deferoxamine along with deferasirox: 119 and deferoxamine along with deferiprone: 429 people). One type of deferasirox is used in 471 subjects (deferasirox: 329, deferasirox along with deferiprone: 23 and deferasirox along with deferoxamine 119).

**Fig. 10:**
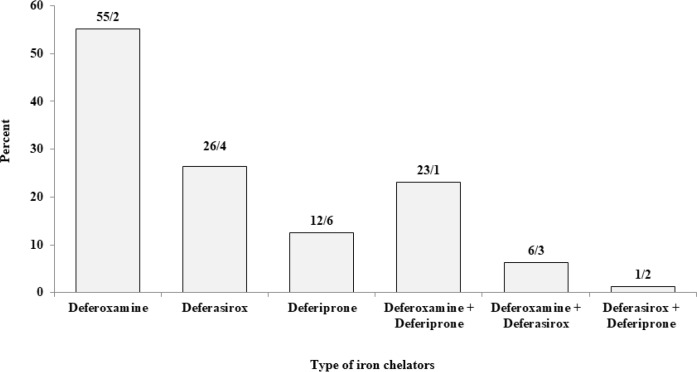
Distribution of 1831 β-thalassemia patients (major and intermedia) according to the type of iron chelators, Mazandaran Province, 2016

Deferiprone was used in 478 patients (deferiprone: 26, deferiprone along with deferasirox: 23 and deferiprone together with deferoxamine: 429 patients). Therefore, 907 (61.4%) and 571 (38.6%) patients used single-agent regimen and combined regimen and also 324 of them did not take any of the iron chelators. Figure 10 shows doses of the iron chelators for all patients reported in various regiments.

**Fig. 11:**
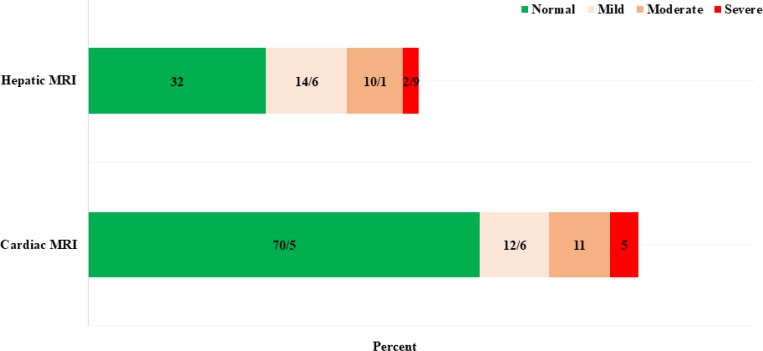
Distribution of 1385 patients with β-thalassemia major in terms of cardiac and liver MRI T2*, Mazandaran Province, 2016

In the whole of the Mazandaran Province, 484 patients (28%) take the hydroxyurea drug and men-proportion is the same (43.3% and 23.3% of patients are in Sari and Amol cities, respectively). Liver MRIT2* examination results were registered in 1340 cases, carried out in 62.2% of cases. The result of the study is characterized by the classification of the severity of liver iron deposition in Fig. 11.

Other widely used drugs are shown in Table 1. Special medications for the treatment of cardiovascular and endocrine diseases and osteoporosis have not been reported in this article.

**Table 1:** Distribution of patients according to the drugs frequently consumed in thalassemic registry, Mazandaran Province, 2016

***Drug name***	***The number of male consumers***	***The number of female consumers***	***Total number of consumers***
Aspirin (80 mg tablet)	516	260	256
Penicillin (500mg tablet)^a^	371	171	200
Folic acid (1 mg tablet)	966	516	450
Calcium carbonate ^b^	831	449	382
Vitamin D3 ^c^	507	276	231
Hydroxyurea ^d^	471	238	233

a 250 mg every 12 h

b Calcium carbonate tablets 500mg ± 200 units Vitamin D daily

c each Pearl Vitamin D, 50,000 units 1 every 3 months

d 500 mg capsules, 1–2 times a day

## Discussion

The prevalence of β-thalassemia population is 1.5% in the world, which is accounts for approximately 60,000 new cases a year (9), declined by between 30,000 and 50,000 people by 2013 (10). About 20,000 thalassemic patients have been registered in Iran by 2003. The prevalence of the disease in the Caspian Sea (Mazandaran, Gilan, and Golestan) and the Persian Gulf (Bushehr, Hormozgan, Sistan and Baluchistan), Khuzestan, Fars, and southern Kerman is higher than in other parts of Iran (9). The prevalence of β-thalassemia is reported to be 10% in these areas (9). At least 1385 patients with β thalassemia major live in Mazandaran Province. There may still patients living in the province who have not registered in any of the state centers. Despite the fact that patients are independent in choosing a doctor and a health center, recording all patients shows a better picture of their situation in the province. Failure to visit or delay in referring patients to the therapeutic system may result in the development of complications caused by overactive bone marrow such as enlargement of the spleen, extramedullary hematopoiesis, osteoporosis, which require more and expensive treatment (2, 11, 12).

The most important indicator and treatment achievement is the increase in the patients’ life span. Survival studies in Iran and other countries show the effect of proper treatment in patients’ life span (13–15). Comparison of the patient’s age in this report with our previous studies conducted in the thalassemia center of Sari (north of Iran) indicates an increase in the average age of patients (16). A remarkable point about the distribution of non-transfusion-dependent patients based on age and gender is that the percentage of men is about half that of women between the ages of 30 and 35, while female/male ratio is almost equal in all other age groups and even in transfusion-dependent patients. The reason for this difference is the risk of non-referral of men born between 1981 and 1986 with non-transfusion-dependent thalassemia. Older women have been forced to come to visit for reasons of the complications, and younger patients have come to the healthcare sector because of cultural change and more attention given to affected girls by their families. Paying attention to measures used to increase referral rate, may lead to more women referrals and the disappearance of this difference. Among the important indicators for treating thalassemic patients, especially those with frequent transfusion, is pre-transfusion amount of hemoglobin. The treatment resources recommend us to always maintain hemoglobin values above 10 gr/dl for controlling and stopping bone marrow hematopoiesis and preventing complications such as facial deformation, biliary stones, and extramedullary hematopoiesis (1–3).

The present study showed that the hemoglobin level in the patients registered in the province was less than 10 gr/dl in 95% of the transfusion-dependent patients. In other words, the blood transfusion rate was lower than that of the patients’ need attributed to the inappropriate cooperation of some patients and, to some extent, the lack of blood samples produced by the blood transfusion organization. Although the blood donation index is 12 units per 1,000 population in developing countries, this index has been reported to be 27 units per 1,000 people in our country. In order to reach advanced countries, the blood donation index should be increased (17). The Blood Transfusion Organization of Iran and the Thalassemia Center of Mazandaran have provided useful activities for improving patients’ services. In order to provide more care for patients during the blood transfusion and recording possible complications, the haemovigilance guidelines have been operational since 2009. They are practiced in all centers of the country. Moreover, filter bags were introduced into the healthcare system since 2005 and used in more than 90% of the PRBC production (more than 2 million units) in 2013 (18). Blood samples that were not subject to filtration process in the organization were passed through the bedside filters before the transfusion step. This action reduces the febrile reactions of the blood (19).

Other indicators of appropriate treatment include iron overload status in thalassemia patients. Although serum ferritin is not a good indicator of total iron in the body, it is measured due to its availability in all centers (20). This index has a better correlation with iron deposition of the liver than cardiac siderosis. Liver MRI is the best indicator having strong correlation with the iron burden of the patient’s body (21, 22). Cardiac and liver MRI was performed in 62.2% of patients in Mazandaran Province, which is a good proportion. In the recorded data, only about 2.9% and 5% of the iron deposition in the liver and heart was severe. This indicator is also used to start iron chelators for non-transfusion-dependent patients; in this way, the average iron deposition in the liver is the indication of initiation of iron chelator (2, 7, 11). Another important point is that there is currently no possibility of examining the heart and liver MRI in Mazandaran Province, while the vast province of Mazandaran needs at least 3 centers for heart and liver MRIT2* for thalassemic patients.

Researches have reported positive and effective effects of hydroxyurea use in thalassemic patients (23, 24). Unfortunately, there is pediatric hematologist/oncologist physician in only one center; therefore, this drug will not be prescribed for the many patients who benefit from it. This drug has been successfully used for transfusion-dependent patients (25).

In addition to iron chelators, patients with thalassemia take different drugs. This article has only investigated general drugs but not specific drugs used for the treatment and control of complications. To prevent thrombosis in patients underwent splenectomy, one tablet of aspirin (80 mg enteric-coated) is prescribed daily (7). Oral penicillin is administered twice a day to prevent opportunistic infections in patients underwent splenectomy (13). Severe folic acid deficiency was previously shown in patients with thalassemia major (26). Consequently, 1 mg of folic acid is prescribed daily for all patients. Considering the high prevalence of vitamin D deficiency in these patients, serum vitamin D testing is carried out for all patients. In addition, for all thalassemic patients, vitamin D deficiency treatment, prescription of vitamin D maintenance dosages and calcium supplement (calcium carbonate 500 mg daily) is recommended (23, 24).

## Conclusion

Thalassemia registry findings are valuable in the management and procurement of drugs for patients. It will also provide managers and experts with access to various levels of information to help them make appropriate decisions.

## Ethical considerations

Ethical issues (plagiarism, data fabrication and/or falsification, double publication and/or submission, redundancy, etc.) have been completely observed by the authors.
